# Qualitative assessment of anal function in swine model for research in colorectal surgery: development of the swine anal function assessment table

**DOI:** 10.1590/acb413726

**Published:** 2026-07-17

**Authors:** Julia Belotto Guaraná, Silvio Henrique de Freitas, Juliana Barreto Salem, Julia Merilis Silva, Fernanda Vieira da Silva, Cinthia Ferreira Lanchotte, Flávio Viera Meirelles, Flávio Henrique Ferreira Galvão

**Affiliations:** 1 Universidade de São Paulo – Faculdade de Zootecnia e Engenharia de Alimentos – Departamento de Medicina Veterinária – Pirassununga (SP) – Brazil.; 2 Universidade de São Paulo – Faculdade de Medicina – Hospital das Clínicas – São Paulo (SP) – Brazil.

**Keywords:** Swine, Rectal Diseases, Transplantation

## Abstract

**Purpose:**

The porcine model is widely used in preclinical studies of anorectal function due to its anatomical and physiological similarity to humans. However, appropriate behavioral and functional evaluation methods of anorectal segment are still lacking.

**Methods:**

This study introduces the swine anal function assessment table (SAFAT), a standardized qualitative tool developed to evaluate anorectal function. Six male Landrace × Large White pigs underwent evaluation following anorectal transplantation over up to one year.

**Results:**

The functional parameters assessed included cleanliness, fecal distribution, stool consistency, anal tone, anal skin sensitivity, anal reflexes, and other ones. Each indicator was rated using semi-quantitative scales based on behavioral observations.

**Conclusion:**

SAFAT was effective for behavioral and functional evaluation after anorectal transplantation and may improve the reproducibility and reliability of functional evaluations in swine models of major anorectal surgeries, supporting translational research in colorectal treatments.

## Introduction

Anorectal disorders such as hemorrhoids, fissures/fistulas, fecal incontinence, anorectal cancer, and other ones are common diseases among the general population. These conditions cause physical pain, worry, and discomfort, affecting the quality of life to different degrees, impacting social, work, and family aspects^1,2^.

Usually, the prevalence of anorectal dysfunction is higher than that observed in clinical practice, as patients may avoid seeking medical care due to embarrassment^3^. Besides, these conditions can be costly to manage, placing risk for debilitating and expensive complications, highlighting their fundamental importance to both healthcare systems and society^4,5^.

Considering this, we have been carrying out in our laboratory extensive translational studies year after year seeking to improve or develop translational models to improve surgical treatments in coloproctology such anorectal transplantation^6-9^. We believe that anorectal transplantation would be the ideal treatment for complex anorectal disfunction or permanent colostomy, mainly for patients with indications for clinical intestinal or multivisceral transplantation since they would be already receiving specific immunosuppression to avoid intestinal graft rejection^10^.

The porcine model has played a critical role as the preferred model for pre-clinical studies of intestinal disease treatments and therapeutic innovations due to their resemblance to human anatomy, physiology, immunology, and disease pathogenesis^11,12^. Besides being developed as a model on their merit, the porcine pre-clinical models have several advantages over other models such as rats, primates, and dogs^9,13,14^.

The small size of rodents makes it difficult to model and advance surgical and endoscopic techniques^12^. Compared to primates, pigs have short generation times, large litter sizes, and well-studied and easily editable genomes^15,16^. Also, pigs face a lower ethical barrier and are more popularly accepted as experimental animals compared to dogs and primates^16^, or even serving as organ donor in the recent clinical trials of heart, kidney, and liver xenotransplantation using transgenic swine^17^.

Even though promising pig models have been developed in recent years^12,15^, well-established porcine models and better evaluation approaches are still missing, as featured by Evers et al.^18^ in a systematic review that reinforces a need for better behavioral assay assessing fecal continence.

This article presents a standardized qualitative table for swine anal function assessment (SAFAT), developed to systematically evaluate sphincter recovery in swine undergoing surgical procedures with potential impact on lower gastrointestinal tract function. The standardization was based on clinical observations of swine during the long-term postoperative period following anorectal transplantation (ART), considering behavioral, motor, sensory, and physiological parameters relevant to anorectal function.

## Methods

The SAFAT was developed in parallel with a swine and canine models to study ART^9,14,19^ and was based on systematic observations and evaluations conducted during long-term postoperative period in both experimental animals (transplanted and control normal animals—non-transplanted, with preserved function).

### Animals and Ethical Committee

Six healthy swine, males, Landrace × Large White hybrids, weighing between 30 and 40 kg (32,9 ± 4,85), were included in the study. Four swine (TARS1-TARS4) were subjected to autologous ART procedure and evaluated postoperatively for up to one year, and two swine (C1-C2) were controls for the experiment and underwent the same evaluations as the other swine, except for the surgical procedure.

This study followed the guidelines of the International Council for Laboratory Animal Science and the Brazilian National Council for the Control of Animal Experimentation. Swine were placed in appropriate pens, had free access to water, and were fed a properly balanced, and portioned diet coherent for their stage of life. This study was approved by Ethical Committee of the Medical School (no. 1,907/2023) and the Faculty of Animal Science and Food Engineering (no. 5,414,100,423) of the Universidade de São Paulo (USP).

### Anesthesia, surgical procedure, and acute postoperative period

The same anesthesiologists and surgical team conducted all procedures as described by Salem et al.^9^. Acute postoperative clinical data were collected daily for 14 days until veterinary discharge; although not relevant to this study, they ensured adequate recovery. Observations for the development of the SAFAT began after discharge and continued throughout the late postoperative period (day 15 to one year).

### Late post-operative care and assessment

Animals were housed at the swine facility at USP’s Faculty of Animal Science and Food Engineering, in pens measuring 4.75 × 2.40 m, with 60% of the area covered. Feed was provided in meal form, and feeding management followed the age-specific feed quantity chart (g/age group) established by the PUSP-FC swine facility. The animals were weighed weekly to monitor and ensure adequate weight gain.

Clinical parameters of control and experimental swine were monitored for up to 12 months. A team of three researchers conducted the assessments to ensure data accuracy and reliability. Daily observations included general health status, welfare, and water and feed intake. Body weight was recorded in the morning before the first feeding, as recommended for accurate weight assessment without interference from gastrointestinal contents.

Other clinical parameters were assessed weekly, due to their minimum variation daily. Researchers tested and included these parameters in the table based on behavioral patterns, intestinal habits, and stimulus responses observed in animals without surgical intervention in the anal and perianal region, compared to those subjected to ART throughout the experiment.

The functional assessment was based on the following parameters: animal cleanliness, fecal distribution in the pen, stool consistency, gastrocolic reflex, involuntary flatulence, anal hypotonia or tone, cutaneous sensitivity of the anoderm, sensitivity response sites, anal sphincter contraction, and strength of sphincter contraction during digital examination.

Animals showing clinical signs of severe complications such as peritonitis, necrosis, complex fistulas, intestinal obstruction, weight loss exceeding 25% of their original or expected weight for their age group, or other alterations judged by the researchers to cause unacceptable pain and discomfort were eligible for euthanasia and necropsy at any point during the study, following an appropriate protocol.

## Results

Animal cleanliness was assessed by visual inspection, considering soiling in the anal, perianal, and gluteal regions. Based on the extent of soiling, scores ranged from 0 to III. Fecal distribution in the pens was also evaluated visually and classified from 0 to II according to feces location (throughout the entire pen, in more than one specific area, or in a single specific area). Stool consistency was assessed visually and scored using the Bristol Stool Scale (0 to 7).

The gastrocolic reflex was assessed by visually assessment the animal during feeding and inspecting whether defecation occurred while eating. This parameter was classified using a dichotomous response (yes/no). The presence of involuntary flatulence and anal hypotonia or tone were evaluated using the same method.

Sensitivity testing of the anoderm was performed using a blunt needle. Animals were stimulated along the entire anal border, and responses were graded from 0 to III based on response intensity. Positive sensitivity responses included tail movement, signs of discomfort, and avoidance of the examiner.

Anal contour areas with greater or lesser responsiveness to cutaneous sensitivity testing were determined by marking the region using a clockface pattern. Responsive areas were described in a clockwise direction according to the zones in which positive sensitivity responses were observed.

Anal sphincter contraction was assessed through visual observation also by stimulating the animal with a blunt needle both inside and outside the surgical scar line along the anal border. Contraction intensity was graded using a cross-based scale (+ to ++++), with control group animals serving as the reference for maximum contraction strength.

Anal sphincter contraction strength was graded through digital rectal palpation. The examiner inserted a finger approximately 2 to 2.5 cm into the swine’s anal canal and, combined with stimulation using a blunt needle, subjectively assessed the sphincter contraction according to a cross-based scale (+ to ++++), using the contraction strength of control group as the maximum reference.

The evaluated indicators resulted in the SAFAT (Table 1), and the authors suggest using an individual spreadsheet to monitor the assessment progression for each animal (Table 2).

**Table 1:** Swine anal function assessment table (SAFAT).

Parameter	Assessment criteria	Score
Animal cleanliness level	Soiling of the anal, perianal, and gluteal regions	0: Clean, I: Slight soiling, II: Moderate soiling, III: Heavy soiling
Feces distribution in the pen	Visual assessment of feces distribution	0: Feces distributed throughout the pen (no control); I: Feces distributed in two or more particular locations within the pen (corners); II: Feces concentrated in a single particular location within the pen
Feces consistency	Visual assessment	Bristol stool scale: 0 to 7
Gastrocolic reflex	Observation of defecation during feeding	0: No defecation, I: Defecation
Involuntary flatulence	Observation during assessment	0: absent, I: present
Hypotonia or tone	Observation during assessment	H: hypotonia, T: tone
Sensitivity of anoderm from internal to surgical border	Test with blunt needle	+: Absence of reflexes to sensitivity test with blunt needle, ++: Mild reflexes to sensitivity test with blunt needle (slight tail movement), +++: Evident reflexes to sensitivity test with blunt needle, ++++: Intense reflexes (marked tail movement; discomfort; examiner avoidance)
Sensitivity response sites	Test with blunt needle	Describe the regions showing contraction response in a clockwise direction
Anal sphincter contraction	Assessment of contraction during sensitivity test with blunt needle	+: No anal sphincter contraction, ++: Slight anal sphincter contraction during sensitivity tests, +++: Evident anal sphincter contraction during sensitivity tests, ++++: Anal sphincter contraction without stimulus (e.g., during eating or grunting)
Anal sphincter contraction strength	Subjective assessment during digital palpation combined with blunt needle test	+: No contraction, ++: Weak contraction, +++: Moderate contraction, ++++: Strong contraction
Others	Noteworthy descriptions	-

Source: Elaborated by the authors.

**Table 2:** Swine anal function assessment table spreadsheet.

Date	Weight	Cleanliness level	Feces distribution	Feces consistency	Gastrocolic reflex	Involuntary flatulence	Hypotonia or tone	Sensitivity of the anoderm	Sensitivity response sites	Anal sphincter contraction	Anal sphincter contraction strength	Others
**_/_/_**										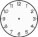		
**_/_/_**										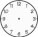		
**_/_/_**										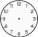		
**_/_/_**										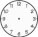		
**_/_/_**										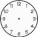		
**_/_/_**										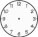		
**_/_/_**										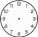		

Source: Elaborated by the authors.

## Discussion

Fecal continence depends on the coordinated action of the sympathetic, parasympathetic, and somatic nervous systems on the rectum and anus^20,21^. It involves the central nervous system, the enteric nervous system, and the spinal defecation center, combining voluntary and involuntary components to control both the sphincters and other pelvic floor muscles^22-25^. However, the defecation process remains poorly understood, making it difficult to determine the relative contribution of each anatomical and neural mechanism involved, as well as voluntary control^26^. For this reason, a multimodal analysis and a combined approach are essential for the assessment of anorectal function.

Each parameter included in this analysis aimed to evaluate specific clinical indicators that reflect the integrity of the diverse components involved in maintaining continence. As with any clinical assessment, it should involve with multiple evaluators, preferably blinded, to ensure objectivity and reliability. Moreover, it is essential to complement these clinical evaluations with other approaches, such as imaging studies, specialized functional tests, and quantitative methods. Combining these methods allows a better comprehension of anatomic and physiological conditions, supporting more robust and reliable research data.

The analysis of defecation habits reveals patterns that reflect not only intestinal function and sphincter control but also animal welfare. Swine exhibit a naturally organized eliminative behavior and tend to keep feeding and resting areas clean, defecating and urinating away from these locations from an early age^27,28^. Therefore, when this behavior is maintained or restored, it indicates satisfactory sphincter function.

However, Nannoni et al.^28^ and Vermeer and Hopster^29^ highlight that environmental variations, such as light and temperature, influence defecation behavior. Under heat stress, swine often change their defecation patterns and may lie on their excreta to dissipate heat. Additionally, aging and weight gain in swine are associated with increased pen soiling due to reduced heat dissipation efficiency in heavier animals and increased body density per square meter^30,31^.

These factors also influence the observation of the animal soiling level parameter, since swine tend to lie on their excreta under heat stress or thermal discomfort^28,29^. Therefore, excessive soiling or defecation in the resting area may indicate not only sphincter dysfunction but also thermal discomfort, space limitation, or environmental stress^32,33^, suggesting that these parameters should be assessed with caution in uncontrolled environments.

Evaluating fecal appearance using the Bristol stool scale is a standardized approach to assess gastrointestinal function and monitor intestinal motility^34^. This scale has been widely used, translated, validated, and culturally adapted in many countries^35^. Although originally developed for evaluating fecal consistency in humans, it has also been applied to other species, including swine, due to the anatomical and physiological similarities in their gastrointestinal tracts^36-38^. Although the Bristol stool scale has not been formally validated for swine, it serves as a descriptive and objective tool, helping to standardize clinical evaluations, reduce subjectivity, and enable comparisons between experimental groups^37^.

The gastrocolic reflex is a physiological response to gastric distension following food intake. It is mainly mediated by the vagus nerve, which increases colonic motility to aid in stool evacuation^38,39^. This process involves both central and peripheral neural pathways and coordinated enteric circuits. The absence, delay, or involuntary defecation during this reflex may signal altered colonic neuromotor conduction or sphincter dysfunction, affecting the animal’s ability to control evacuation.

The transient relaxation of the internal anal sphincter, triggered by the rectoanal inhibitory reflex, enables the upper anal canal to differentiate between gases, liquids, and solids^40,41^. Monitoring involuntary flatus might provide insight into the functional integrity of the rectum and indicate anorectal sensory dysfunction or altered motor responses of the internal or external sphincters^42^. However, this parameter should not be considered alone, since the connection between involuntary gas loss and true fecal incontinence remains unclear^43^.

Anal tone is a reliable criterion for evaluating anorectal functionality. Anal tonicity is basally maintained by the involuntary contraction of the internal anal sphincter (IAS), which accounts for up to 85% of the resting anal pressure^25,26^. The external anal sphincter (EAS), on the other hand, contributes to anal pressure during periods of rectal distension through a voluntary mechanism controlled by the somatic nervous system^20,26^. Studies have shown that, even after the transection of the pudendal nerves in experimental models, anal tone and IAS function are preserved, since the involuntary contraction of this muscle does not depend on pudendal innervation^23,25^.

The pudendal nerves play a critical role in the voluntary innervation of the EAS, reaching this muscle predominantly at the 2 and 10 o’clock positions, according to an imaginary clockface surrounding the anal margin^19^. Stimulation of the anal border using a blunt needle enables evaluation of cutaneous sensory recovery following surgical procedures in the anal and perianal regions. Given the anatomical trajectory of the pudendal nerves, site-specific sensitivity assessment was incorporated as a parameter in the SAFAT to investigate localized neuromuscular responses. The complementation of the blunt needle exam with digital rectal palpation as proposed by SAFAT analyses the recovery of anal contractility, and the grading of sphincter contraction strength through sphincter response to tactile stimuli. These parameters primarily aim to assess the neuromuscular function of the anal sphincter and the functionality of the anorectal segment.

When using SAFAT, researchers should expect to obtain valuable data on the clinical assessment of anal function in swine, but they must also recognize its inherent limitations. Environmental and physiological factors—including thermal stress, available space, age, and weight gain—can influence elimination behaviors and confound parameter interpretation. Additionally, the subjectivity inherent in clinical and behavioral evaluations, even when conducted by a trained team, can introduce biases. Therefore, SAFAT should be applied alongside complementary methods, such as imaging, specialized functional tests, and quantitative analyses, to ensure a precise evaluation of anorectal function.

## Conclusion

The SAFAT was developed to be a valuable tool for monitoring anal function in swine experimental models, enabling consistent and standardized clinical data collection. The range of clinical responses recorded offers important insights for colorectal research by reflecting neuromuscular recovery and anal segment functionality.

## Data Availability

The data that support the findings of this study are available from the corresponding author, upon reasonable request.
